# Dominance of recombinant cotton leaf curl Multan-Rajasthan virus associated with cotton leaf curl disease outbreak in northwest India

**DOI:** 10.1371/journal.pone.0231886

**Published:** 2020-04-22

**Authors:** Kajal Kumar Biswas, Utpal Kumar Bhattacharyya, Supratik Palchoudhury, Nenavath Balram, Anil Kumar, Rupesh Arora, Satish Kumar Sain, Pradeep Kumar, Ravi K. Khetarpal, Amitava Sanyal, Pranab Kumar Mandal

**Affiliations:** 1 Advanced Centre for Plant Virology, Division of Plant Pathology, ICAR-Indian Agricultural Research Institute, New Delhi, India; 2 Chaudhary Charan Singh Haryana Agricultural University, Haryana, India; 3 Regional Research Station, Punjab Agricultural University, Punjab, India; 4 ICAR-Central Institute for Cotton Research, Regional Station, Haryana, India; 5 Agricultural Research Station, Swami Keshwanand Rajasthan Agriculture University, Rajasthan, India; 6 Asia-Pacific Association of Agricultural Research Institutions, Bangkok, Thailand; 7 Sumitomo Chemical India Pvt. Ltd., New Delhi, India; 8 ICAR-National Institute for Plant Biotechnology, New Delhi, India; Oklahoma State University, UNITED STATES

## Abstract

Cotton leaf curl disease (CLCuD), caused by whitefly (*Bemisiatabaci*) transmitted single-stranded DNA viruses belonging to the Genus, *Begomovirus* (family, *Geminiviridae*) in association with satellite molecules; is responsible for major economic losses in cotton in three northwest (NW) Indian states Haryana, Punjab, and Rajasthan. Annual CLCuD incidences during 2012 to 2014 were estimated to be 37.5%, 63.6%, and 38.8% respectively. Cotton leaves were collected from symptomatic plants annually for three years and subjected to DNA isolation, followed by rolling circle amplification (RCA), cloning, and DNA sequencing of apparently full-length begomoviral genomes and associated betasatellites and alphasatellites. Among the thirteen CLCuD-begomoviral genomes recovered, eight were identified as *Cotton leaf curl Multan virus*-Rajasthan (CLCuMuV-Ra), one as -Pakistan (PK) and another as -Faisalabad (Fai), whereas, three were as *Cotton leaf curl Kokhran virus*-Burewala (CLCuKoV-Bu), indicating that CLCuMuV-Ra was the most prevalent begomovirus species. Five of the eight CLCuMuV-Ra sequences were found to be recombinants. The CLCuMuV-Ra- associated satellites consisted of *Cotton leaf curl Multan betasatellite* (CLCuMB), and *Gossypium darwinii symptomless alphasatellite* (GDarSLA), and *Croton yellow vein mosaic alphasatellite* (CrYVMoA). The second most abundant helper virus species, CLCuKoV-Bu, was associated with CLCuMB and GDarSLA.

## Introduction

Cotton (*Gossypium hirsutum*), the most important commercial crop, produces 27% of world’s cotton occupying the largest production area of 11.9 million hectares (Mha) in India comprising 38% of total land devoted to cotton cultivation worldwide [1]. The cotton leaf curl disease (CLCuD) has become a major economic constraint to cotton production in the Haryana, Punjab and Rajasthan states in northwestern (NW) India accounting for 1.1 Mha [2–4]. The CLCuD is caused by whitefly (*Bemisiatabaci*)-transmitted monopartite (~2.7 kb ssDNA-A) begomoviral species in association with betasatellite (~1.35 kb ssDNA) and alphasatellite molecules (~1.4 kb ssDNA) that results in the world’s losses to the cotton [4–7]. The CLCuD associated begomovirus genome encodes the coat protein (CP; V1 ORF) and V2 protein on the virion-sense, and the replication-associated protein (Rep; C1 ORF), transcriptional activator protein (TrAP; C2 ORF), replication enhancer protein (REn; C3 ORF) and C4 protein on the complementary-sense strand [5,8]. The betasatellite molecule has three major features; a βC1 gene, an A-rich region, and the satellite conserved region containing a stem-loop structure [9]. The βC1 gene, located in the complementary-sense strand, has a role in symptom induction and acts as a suppressor of both the transcriptional gene silencing (TGS) and the post transcriptional gene silencing (PTGS) [10–13]. The βC1 enhances the viral genome levels and involves in virus movement in plants [5,14]. The alphasatellite molecules possess three conserved regions; (i) a replication-associated protein (Rep), a rolling-circle replication initiator, (ii) an A-rich region, and (iii) a stem-loop structure [15]. The Rep gene of alphasatellite is involved in overcoming host defense by suppressing both TGS and PTGS [16,17]. Alphasatellite has a role in attenuating or exacerbating symptoms and reducing betasatellite accumulation in plants [18–20].

High genetic diversity of CLCuD-begomovirus species has been documented in cotton growing areas in India and Pakistan [21,22]. More than nine CLCuD-begomoviruses species have been reported to be associated with the CLCuD in the Indian subcontinent [23]. The most widespread and core CLCuD-begomoviruses are *Cotton leaf curl Alabad virus* (CLCuAlV), *Cotton leaf curl Kokhran virus* (CLCuKoV), and *Cotton leaf curl Multan virus* (CLCuMuV) [7,21,22,24,25]. The non-core CLCuD-begomoviruses are *Cotton leaf curl Bangalore virus* (CLCuBaV), *Cotton leaf curl Gezira virus* (CLCuGeV), *Okraenation leaf curl virus* (OEnLCV), *Papaya leaf curl virus* (PaLCuV), *Tomato leaf curl Bangalore virus* (ToLCuBaV), *Tomato leaf curl New Delhi virus* (ToLCNDV), and occasionally associated with the CLCuD complex in the Indian subcontinent [21,24,26,27]. In addition, one mastrevirus, *Chickpea chlorotic dwarf virus* (CpCDV) has been reported in association with leaf curl symptoms in cotton in Pakistan [28]. The *Cotton leaf curl Multan betasatellite* (CLCuMB) is the only betasatellite species detected in association with the CLCuD complex in the Indian subcontinent [7,21–22,25,29,30]. A number of different alphasatellites, *Cotton leaf curl Multan alphasatellite* (CLCuMuA), *Cotton leaf curl Lucknow alphasatellite* (CLCuLuA), *Gossypium darwinii symptomless alphasatellite* (GDarSLA) and *Gossypium mustilinum symptomless alphasatellite* (GMusSLA) have been reported to be associated with the CLCuD complex in this subcontinent [7,21–22,25,29,31]. The recombination phenomenon is a major driving force in the evolution of begomovirus genomes [32–35] including CLCuD-begomoviruses [7,25,36]. The CLCuMB sequence has also been reported to be a recombinant [37] and this recombinant betasatellite has role in resistance-breaking in cotton in Pakistan [37,38].

The CLCuD was first recognized during the 1960s in Pakistan, but the outbreak occurred in and after the year 1990 near Multan, and then spread rapidly to most of the cotton growing areas in Pakistan [21]. In India, CLCuD was first noticed at Indian Agricultural Research Institute (IARI), New Delhi in the year 1989 [39], and one farmer’s field in Sri Ganganagar of Rajasthan state in 1993, and Punjab and Haryana state in 1994 [40]. After that CLCuD became a major threat to cotton cultivation in NW India [40,41]. The damage caused by CLCuD during the years of 1997 to 2006 in India was managed due to the cultivation of resistant cotton varieties and intensive measures to control the insect vector whitefly, and weeds. However, the complete replacement of varieties with new Bt-cotton hybrids after the year of 2007 changed the disease scenario. Most of the irrigated cotton in NW India was infected by CLCuD-begomovirus showing about 97% incidence with 53.6% yield loss in some farmer’s field [3,4]. Afterward, the CLCuD is increased year by year and recently emerged as a devastating disease in entire 1.1 Mha cotton growing areas of NW India.

In past three decades, in the Indian subcontinent, CLCuD epidemics struck twice; first (i) “Multan epidemic” in Pakistan between the years of 1989 to 1999, and in NW India between 1997 to 2005, and second (ii) “Burewala epidemic” in Pakistan between the years of 2002 to 2014 and in NW India between 2009 to 2010 [4,21]. Multan epidemic is reported to be caused by three begomovirus species, CLCuAlV, CLCuKoV and CLCuMuV [42]. After the Multan epidemic, the cotton productivity was restored by developing resistant cultivars, but during the years 2002–2003 and onwards, all the resistant cultivars developed became susceptible to CLCuD and it was happened due to appearance of Cotton leaf curl Burewala virus (now known as CLCuKoV-Burewala strain) [4,38,43]; this was the start of the second epidemic of CLCuD, as Burewala epidemic [6]. Currently, CLCuMuV-Rajasthan (CLCuMuV-Ra) and CLCuKoV-Burewala (CLCuKoV-Bu) are prevalent begomoviruses causing CLCuD in NW India [4,7,29–30], whereas, CLCuMuV-Pakistan (CLCuMuV-PK), CLCuMuV-Ra and CLCuKoV-Shadadpur (CLCuKoV-Sha) are prevalent in Pakistan [22].

CLCuD incidence was found to be quite high as 51.3–57.8% in all the surveys made from the years of 2012 to 2014 in Punjab state (India), but in Haryana and Rajasthan states significant variations of 32.7 to 77.5% and 8.9 to 59.2% respectively were observed. These observations suggest the changes in CLCuD-begomovirus genetic composition in NW India. To explain the basis for the suspected changes in the etiology of CLCuD-begomoviruses and satellite molecules associated with the CLCuD outbreak, the infected cotton leaf samples collected from different areas were analyzed based on molecular approaches for determination of the specific begomoviruses, and their satellite molecules, those are most prevalent in NW India.

## Materials and methods

### Estimation of disease incidence and collection of cotton plant and whitefly samples

A survey was made in the years of 2012, 2013, and 2014 to estimate CLCuD incidence in cotton growing areas of NW India. Fatehabad, Hisar, Rohtak and Sirsa districts of Haryana state; Bathinda, Faridkot, Fazilka and Mansa districts of Punjab state; and Hanumangarh and Sri Ganganagar districts of Rajasthan state were considered for the survey. Cotton plants exhibiting vein thickening, leaf curling, leaf cupping, and leaf enations were considered to be the symptoms of the CLCuD. The percent disease incidence (PDI) was estimated using a standard method based on the number of plants infected compared to the number of plants counted ranging from 100–200 randomly per field site multiplied by 100 with three replications. Leaves of symptomatic cotton plants were randomly collected, placed in labelled polythene bags, transported to the laboratory, and stored at 4°C prior to the isolation of DNA. The adult whiteflies were collected from the CLCuD affected cotton plants with the help of an aspirator from three areas, (i) Agricultural Research Station (ARS), Swami Keshwanand Rajasthan Agricultural University (SKRAU), Sri Ganganagar, Rajasthan; (ii) Regional Station (RS), Central Institute for Cotton Research (CICR), Sirsa, Haryana; and (iii) Regional Research Station (RRS), Punjab Agricultural University (PAU), Bhathinda, Punjab (India). The whiteflies were placed in the collection tubes containing 95% ethanol and brought to the laboratory for molecular assay.

### Source of whitefly for pathogenicity test

The whitefly used for virus transmission in this study was initially obtained from the eggs laid on bottle gourd (*Lagenariasiceraria*cv. Pusa Naveen) leaves of the Experimental Farm, CICR, Sirsa, Haryana. The adult whiteflies emerged from the pupae were transferred on the healthy cotton (cv. RST-9) and bottle gourd (cv. Pusa Naveen), reared and multiplied in the insect-proof chamber for 4–5 generations. A homogeneous population of whiteflies was used for the virus transmission studies. Health of whitefly populations was confirmed by PCR test using the primer pair, C3F (5'AATTATGTCGAAGCGAGCTG3') and G1R (5'TAATATCAATTCGTTACAGAG3') [36] targeting the complete CP gene (771 bp) of CLCuD-begomovirus genome in randomly sampled whiteflies reared on cotton.

### Ethics statement

No permits are required for the collection of cotton plant and whitefly samples from the farmer’s field in cotton growing areas of NW India surveyed.

### Infectivity test

Infectivity of the CLCuD was tested through adult whitefly inoculation onto susceptible cotton plant in the greenhouse. The methods of the maintenance of healthy whitefly, and inoculation of the virus through whitefly, developed by Godara et al. [44] were used in the study. Infectivity of CLCuD affected cotton samples randomly collected from different areas of NW India was tested using susceptible cotton cv. RST-9. Tender symptomatic twigs of cotton samples were used as a source of virus. The healthy whiteflies were collected from the culture stock in a small collection cage and transferred to the symptomatic cotton twig for the acquisition access period (AAP) of 24h. After completion of AAP, the viruliferous whiteflies were transferred to the healthy test cotton plants for the inoculation acquisition period (IAP) of 24h. After AAP, the viruliferous whiteflies were collected using an aspirator (glass tube fitted with nylon pipe and muslin cloth). Cotton seedlings at two-leaf stage were used for inoculation. Eight to ten viruliferous whiteflies per healthy cotton plant were released and covered with small plastic cages which covered each plant individually. After IAP, the whiteflies were killed by spraying insecticide, Pyriproxifeen 10% EC (Lano) @ 0.2%. The whitefly inoculated plants were kept in an insect-proof greenhouse for six weeks and observed regularly for symptom development. The leaves of inoculated symptomatic and non-symptomatic plants were processed for PCR analysis for confirmation of the CLCuD-begomovirus infection.

### Total DNA isolation from plant and whitefly samples

Total plant DNA was extracted from 100 mg of infected cotton leaf tissue using the Cetyltrimethylammonium bromide (CTAB) method [45]. The DNA was visualized by electrophoresis in a 1% agarose gel in 1X TAE buffer (pH 8.0) and quality was evaluated by the spectrometry using a Nanodrop (Thermo Fisher Scientific Inc, Waltham, USA). Leaves of two healthy cotton plants maintained in the insect-proof greenhouse were taken for isolation of total plant DNA and used as controls throughout the experiments.

For isolation of the total DNA from whitefly population, a pool of 10 numbers of adult whiteflies was taken from the collection tube and the ethanol was air-dried for 2–5 min on a piece of filter paper. Whiteflies were placed in 1.5 ml micro-centrifuge tube and the total DNA was extracted using Nucleo-pore DNA Sure Tissue Mini Kit (Genetix Biotech Asia Pvt. Ltd. India, Cat No. NP-61305) according to the manufacturer’s instructions. The total DNA was eluted in 100 μL pre-warmed buffer BET (70°C) in a 1.5 ml microcentrifuge tube.

### Cloning of full-length genome of CLCuD-begomovirus, satellite molecules and mtCOI gene of whitefly

The full-length circular genomes of begomovirus and satellite molecules were amplified from the infected cotton plant samples through rolling circle amplification (RCA) using phi29 DNA polymerase [46]. The concatamers were incubated with 1U of the restriction enzymes *Bam* HI, *Eco* RI, *Hind* III, *Sal* I and *Xba* I separately to release the full-length sequence of begomovirus (~2.7 kb) and satellite molecule (1.3–1.4 kb). The digested RCA products were eluted from 1% agarose gel, column purified (Qiagen, Maryland, USA: Cat No. 28115), ligated to pUC18 (Thermo Fisher Scientific, MA, USA), and transformed into competent *E*. *coli* DH5α cell using the standard protocol [47]. The positive clones were selected from the transformation Petri-plates, cultured and the plasmid DNA was purified (Real Biotech Corporation, Taipei, Taiwan, Cat No. QPD100), and sequenced in an ABI 3130 automatic sequencer (Chromous Biotech Pvt. Ltd., Bangalore, India) using vector-derived M13 forward and reverse primers. Additional primers were designed based on the Sanger sequencing results and used for primer walking to obtain the complete genome sequence of the begomovius and satellite molecules, respectively.

The total DNA extracted from whitefly was used as the template for amplification of the partial mitochondrial cytochrome oxidase I (mtCOI) gene of whitefly using allele specific primers; CI-J Forward: 5'TTGATTTTTTGGTCATCCAGAAGT3' and TL2 Reverse: 5'TCCAATGCACTAATCTGCCATATTA3' [48,49]. The PCR amplicons were cloned in T&A cloning vector system (RBC, Taipei, Taiwan) and sequenced in an automatic sequencer (ABI 3130, Chromous Biotech Pvt. Ltd., Bangalore, India).

### Sequence analysis and phylogenetic relationship

All the DNA sequences of begomoviuses, satellite molecules and mtCOI gene of whitefly were quality-checked against the electropherogram and manually edited. The vector sequences were removed using the Bioedit version 7.1.3 [50]. The coding regions of the virus and satellite DNAs were identified using the NCBI ORF finder (www.ncbi.nlm.nih.gov/orffinder/) and annotated in the context of the expected coding and non-coding regions. All the sequences were searched for similarities using BLASTn tools, and the respective top 10–15 sequence matches available in the GenBank (http://www.ncbi.nlm.nih.gov) were downloaded.

For sequence analysis of the present CLCuD-begomoviruses, full-length genome sequences of other CLCuD-begomoviruses available in NCBI-GenBank were retrieved [4,7,22,29,36] including the recognized CLCuD-begomoviruses, CLCuAlV, CLCuMuV and CLCuKoV [51]. One sequence from each of the *Cotton leaf curl Gezira virus* (CLCuGeV: AF260241) and *Cotton leaf curl Bangalore virus* (CLCuBaV: AY705380) were taken as outgroups. For sequence analysis of the present satellite molecules, other satellite molecules associated with CLCuD-begomovirus and other begomoviruses were retrieved from NCBI- GenBank. To ascertain the genetic group status of whitefly populations, the nucleotide sequences of the partial mtCOIgene of whitefly were analyzed and compared with reference sequences retrieved from the NCBI- GenBank.

For phylogenetic analysis, the sequences were aligned using MEGA, version 6.06 [52] implemented in Clustal W v1.6 [53]. The phylogenetic tree was re-constructed using Neighbor-Joining (NJ) [54] with 1000 bootstrap iterations. To determine the pairwise nucleotide identity, a pairwise matrix was computed using the Sequence Demarcation Tool (SDT) version 1.2 [55].

### Recombination analysis

Recombination analysis was carried out using Recombination Detection Program (RDP) version 4.66 implementing seven algorithms BootScan, Chimera, Geneconv, Maxchi, RDP, SiScan and 3Seq. The default settings were used to establish the Bonferroni-corrected highest acceptable P-value cut-off of 0.05 to identify predicted recombinants [56]. Each event was verified based on a breakpoint distribution plot, and results were compared against the UPGMA phylogenetic trees produced with genetic regions from major and minor parents. The recombination events detected in begomovirus genome by three or more, and in betasatellite and alphasatellite genomes by two or more algorithms were considered to be true recombination events.

## Results and discussion

### Changing Cotton Leaf Curl Disease (CLCuD) scenario in NW India

**Fig 1:**
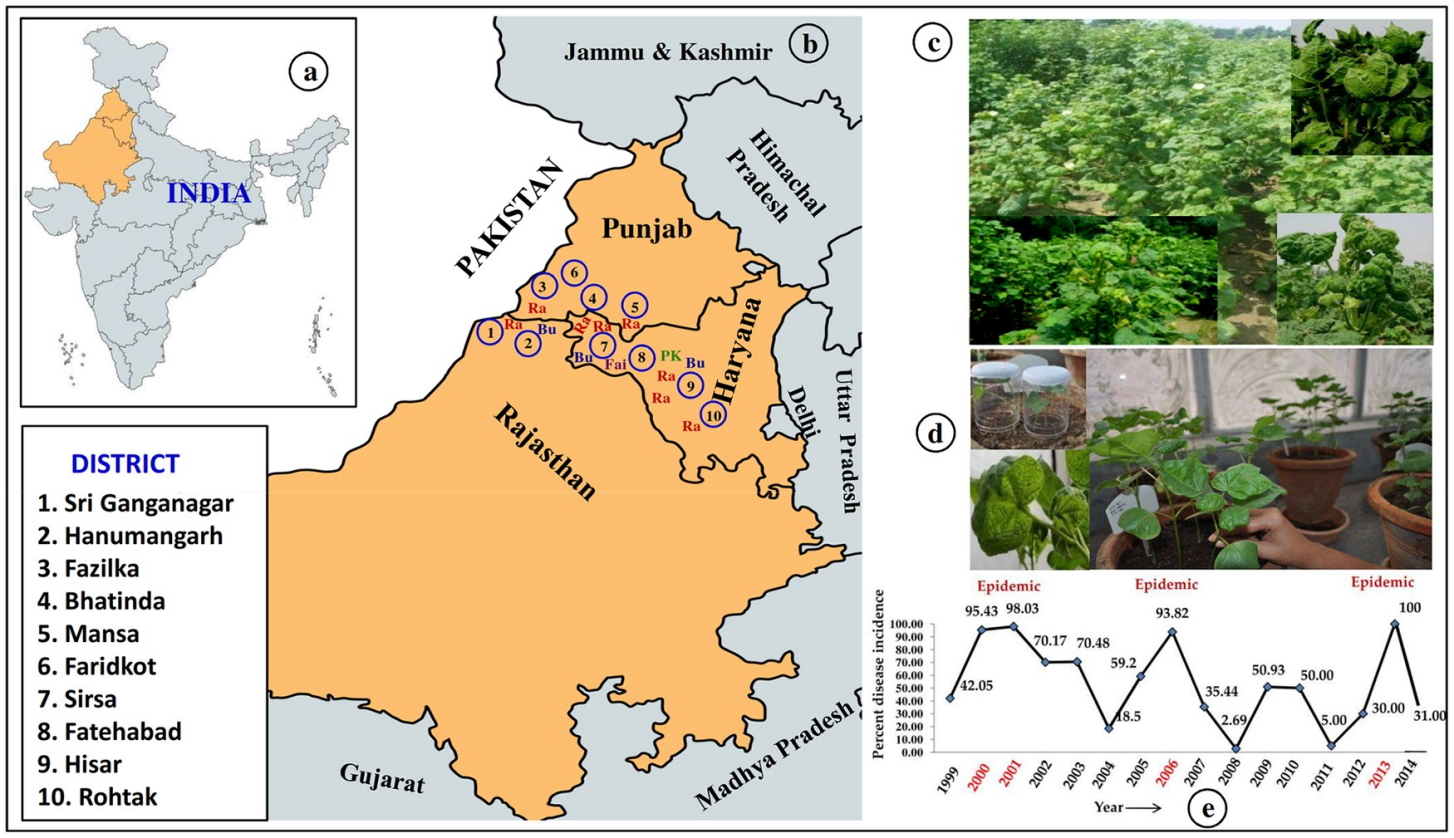
The plate showing cotton growing states in northwest India (a); Different cotton growing areas of Haryana, Punjab and Rajasthan and distribution of CLCuD-begomovirus strains, Ra: CLCuMuV-Ra, Bu: CLCuKoV-Bu, PK: CLCuMuV-PK, Fai: CLCuMuV-Fai strains (b); CLCuD affected cotton exhibiting different kinds of symptoms in the farmer’s field of NW India (c); Infectivity test of CLCuD-begomovirus isolates collected from field through whitefly inoculation (d); Line graph showing the CLCuD incidence of susceptible cotton cv. HS-6 at the experimental field of CICR-Sirsa, Haryana, India from 1999 to 2014 by year (Anonymous, 2016) (e).

The surveys revealed a variation of CLCuD incidence in cotton growing areas of NW India from year to year and area to area (Fig 1). CLCuD incidence was estimated to 32.7, 51.3 and 28.6% in 2012; 77.5, 54.1 and 59.2% in 2013; and 49.6, 57.8 and 8.9% in 2014 in Haryana, Punjab, and Rajasthan states, respectively (S1 Table). Overall disease incidence was recorded to 37.5 in 2012, 63.6 in 2013 and 38.8% in 2014 in NW India. Interestingly, the disease was very high as 77.5, 54.1 and 59.2% in Haryana, Punjab, and Rajasthan, respectively in 2013. Interestingly, CLCuD incidence was observed constantly as high as 51.3–57.8% in all the three years in Punjab. In 2010, CLCuD was also reported very high ranging from 50 to 100% incidence in some districts of Punjab and Rajasthan, but sporadic (0–30%) in Fazilka districts of Punjab and Hanumangarh districts of Rajasthan [4]. The previous and the present data indicated that the etiology of CLCuD-begomovirus complex in NW India is changing from year to year and area to area.

### Whitely cryptic species Asia II 1 is prevalent in NW India and it transmits CLCuD-begomovirus with high efficiency

In the present study, three sequences of the partial mtCOI gene (867 bp) of three whitefly samples, accession no.’s MN329161, MN329162 and MN329163 were analyzed. All the three sequences had maximum of 94–95% nt identity with the sequences of whitefly cryptic speciesAsia II 1-[China: Zhejiang] (AJ867557) (S2 Table) and they clustered in one phylogenetic group along with Asia II 1 (S1 Fig). These data concluded that the present whitefly populations are whitefly cryptic species Asia II 1. Based on the mtCOI sequence analysis, Ellango et al. [57] reported earlier that whitefly cryptic species Asia II 1 is mostly occurred in the cotton growing areas of NW India. This cryptic species Asia II 1 is also reported to be the most abundant and associated with a high CLCuD outbreak in the cotton growing areas of Pakistan [58].

Transmission efficiency of CLCuD-begomovirus by whitefly cryptic species Asia II 1 was estimated using susceptible cotton cv.RST-9 in a greenhouse. Six CLCuD-begomovirus infected cotton samples randomly collected from six cotton growing areas, Fazilka (Faz 14), Hanumangarh (Hmg 14), Hisar (Uf-1), Mansa (Ma14-3), Sirsa (S9) and Sri Ganganagar (SG-14) of NW India were used as source of inoculums for whitefly transmission (Fig 1d). The whitefly inoculated cotton plant induced typical CLCuD symptoms within 10–28 DAI showing transmission efficiency of 80–100% (S3 Table), indicating whitefly cryptic species Asia II 1 is efficient vector for CLCuD-begomovirus in NW India. Recently, the transmission efficiency of CLCuMuV by whitefly cryptic species Asia II 1 has been reported to be very high [59].

### CLCuMuV-Rajasthan strain is predominant begomovirus associated with CLCuD outbreak in NW India

**Fig 2:**
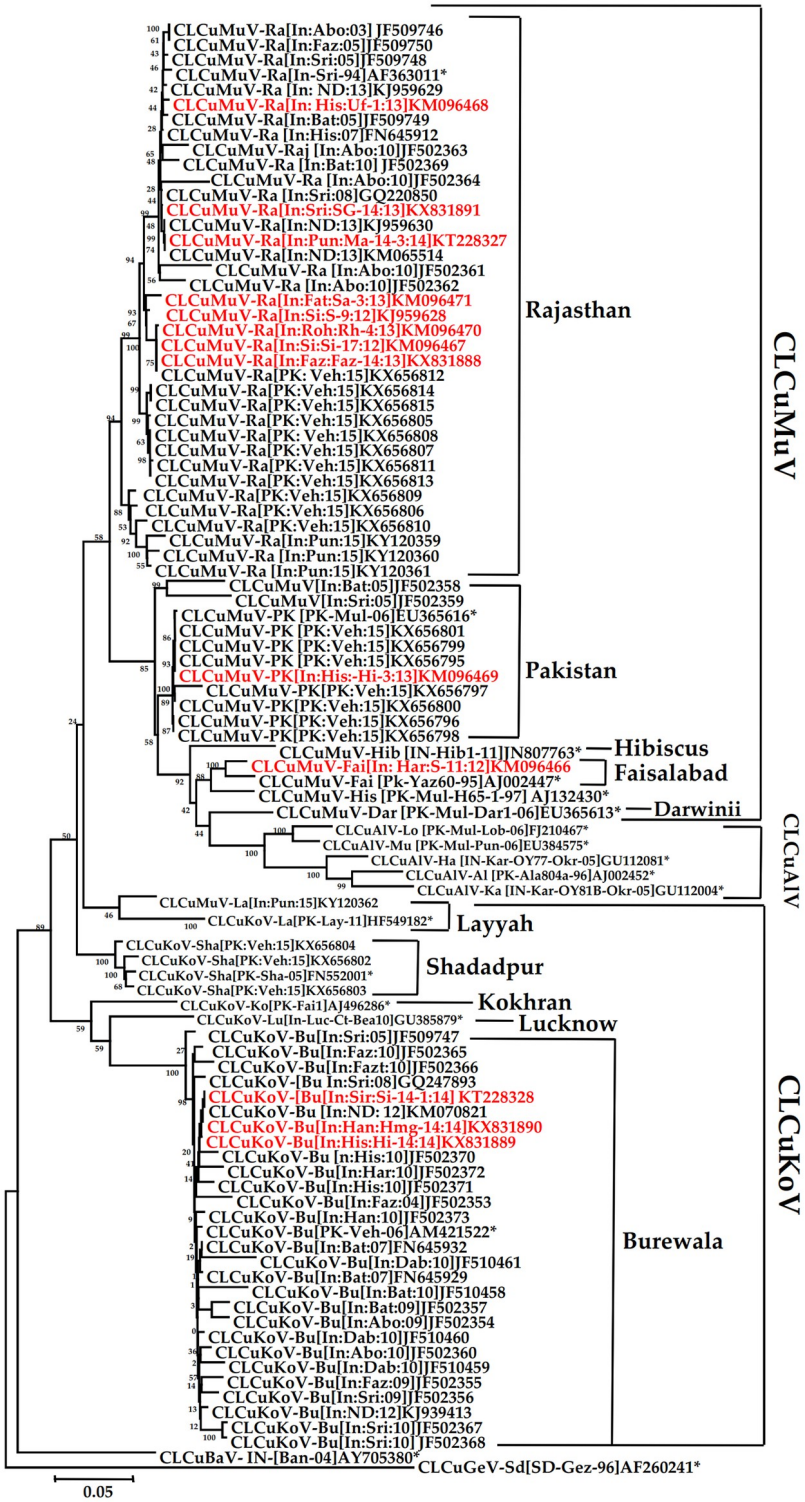
Phylogenetic relationships based on the complete genome of the present CLCuD-begomoviruses with other CLCuD-begomoviruses available in the NCBI-GenBank. The tree was generated using the Neighbor-Joining (NJ) in MEGA 6. The tree was generated 1000 bootstrap values shown next to the branches. The sequences generated in the present study are represented by the red colour font. The sequences of CLCuD-begomovirus strains described by brown *et al*. [52] are highlighted by the star (*). CLCuMuV and CLCuKoV clades and their strains subclades demarcated in the right panel of the figure.

All the thirteen DNA-A sequences obtained from CLCuD-begomovirus infected cotton samples had ~2.7 kb and their genome organization showed seven ORFs, those were typical to the DNA-A component of other CLCuD-begomoviruses (Table 1). All the present DNA-A sequences had 81–100% nt identities among themselves. According to the species demarcation cut-off value of ≥91% nt identity approved by ICTV, they belong to two CLCuD-begomovirus species, CLCuMuV and CLCuKoV. Eight sequences, Faz-14 (KX831888), Ma-14-3 (KT228327), Rh-4 (KM096470), Sa-3 (KM096471), Si-17 (KM096467), S-9 (KJ959628), SG-14 (KX831891) and Uf-1 (KM096468) had close homology (95–100% nt identities) among themselves. These sequences were closely related to CLCuMuV-Ra (AF363011) strain (Table 2) based on the strain demarcation cut-off value of ≥94% given by Brown et al. [51]. Three DNA-A sequences, Hi-14 (KX831889), Si-14-1 (KT228328) and Hmg-14 (KX831890) showed 98–100% nt identities among themselves and similarly based on strain demarcation cut-off value, they were related to CLCuKoV-Bu (AM421522) strain (Table 2). Similarly the sequence, S-11 (KM096466) was related (97% identity) to CLCuMuV-Faislabad (CLCuMuV-Fai:AJ002447) strain and Hi-3 (KM096469) related (cent percent identity) to CLCuMuV-PK (EU365616) strain (Table 2). In the NJ phylogenetic tree analysis, the present DNA-A sequences segregated into four clades; eight sequences, Faz-14, Ma-14-3, Rh-4, Sa-3, Si-17, S-9, SG-14 and Uf-1 grouped with CLCuMuV-Ra; S-11 with CLCuMuV-Fai; Hi-3 with CLCuMuV-PK; and three sequences Hi-14, Hmg-14 and Si-14-1 with CLCuKoV-Bu strains (Fig 2).

**Table 1:** Genomic properties of the DNA A of CLCuD-begomoviruses generated in the present study.

CLCuD sequence	Origin (Year of collection)	Symptom	Associated components	Acc. No	Size (nt)	ORFs (coordinates/nt/aa)						
						V2	V1	C1	C2	C3	C4	C5
Faz-14	Fazilka, Punjab (2013)	DC, UC, LE	CLCuMuV-Ra	KX831888	2752	131-487/ 357/118	291-1061/ 771/256	1510-2598/ 1089/362	1161-1613/ 453/150	1064-1468/ 405/134	2142-2444/ 303/100	75-806/ 732/243
Ma-14-3	Manza, Punjab (2014)	Sev DC, UC, LE	CLCuMuV-Ra	KT228327	2753	131-487/ 357/118	291-1061/ 771/256	1510-2598/ 1089/362	1161-1613/ 453/150	1064-1468/ 405/134	2142-2444/ 303/100	75-806/ 732/243
Rh-4	Kharkhara, Rohtak, Haryana (2013)	DC, UC, Vt, LE	CLCuMuV-Ra	KM096470	2753	131-487/ 357/118	291-1061/ 771/256	1510-2598/ 1089/362	1161-1613/ 453/150	1064-1468/ 405/134	2142-2444/ 303/100	75-806/ 732/243
S-9	CICR,Sirsa, Haryana (2012)	Sev DC, UC, LE	CLCuMuV-Ra	KJ959628	2753	131-487/ 357/118	291-1061/ 771/256	1510-2598/ 1089/362	1161-1613/ 453/150	1064-1468/ 405/134	2142-2444/ 303/100	75-806/ 732/243
Sa-3	Sahanwala, Fatehabad, Haryana (2013)	DC, LE	CLCuMuV-Ra	KM096471	2748	118-474/ 357/118	278-1048/ 771/256	1497-2585/ 1089/362	1148-1600/ 453/150	1051-1455/ 405/134	2129-2431/ 303/100	62-793/ 732/243
Si-17	Moriwala,Sirsa, Haryana (2012)	DC, UC, Vt, LE	CLCuMuV-Ra	KM096467	2752	131-487/ 357/118	291-1061/ 771/256	1510-2598/ 1089/362	1161-1613/ 453/150	1064-1468/ 405/134	2142-2444/ 303/100	75-806/ 732/243
SG-14	Sri Ganganagar, Rajasthan (2013)	UC, Vt, LE	CLCuMuV-Ra	KX831891	2753	131-487/ 357/118	291-1061/ 771/256	1510-2598/ 1089/362	1161-1613/ 453/150	1064-1468/ 405/134	2142-2444/ 303/100	75-806/ 732/243
Uf-1	CCSHAU, Hisar, Haryana (2013)	DC, LE	CLCuMuV-Ra	KM096468	2753	131-487/ 357/118	291-1061/ 771/256	1510-2598/ 1089/362	1161-1613/ 453/150	1064-1468/ 405/134	2142-2444/ 303/100	75-806/ 732/243
S-11	Panjwana, Haryana (2012)	DC, UC	CLCuMuV-Fai	KM096466	2748	117-482/ 366/121	277-1047/ 771/256	1497-2585/ 1089/362	1147-1599/ 453/150	1050-1454/ 405/134	2128-2430/ 303/100	62-793/ 732/243
Hi-3	Ghanakalan, Hisar, Haryana (2013)	UC, LE	CLCuMuV-PK	KM096469	2739	116-481/ 366/121	276-1046/ 771/256	1495-2583/ 1089/362	1146-1598/ 453/150	1049-1453/ 405/134	2127-2429/ 303/100	60-584/ 525/174
Hi-14	Hisar, Haryana (2014)	UC, Vt	CLCuKoV-Bu	KX831889	2759	132-488/ 357/118	292-1062/ 771/256	1505-2596/ 1092/363	1295-1504/ 210/69	1059-1463/ 405/134	2242-2682/ 441/146	283-807/ 525/174
Hmg-14	Hanumangarh, Rajasthan (2014)	DC, LE	CLCuKoV-Bu	KX831890	2759	132-488/ 357/118	292-1062/ 771/256	1505-2596/ 1092/363	1295-1504/ 210/69	1059-1463/ 405/134	2242-2682/ 441/146	283-807/ 525/174
Si-14-1	Sirsa, Haryana (2014)	DC, UC, LE	CLCuKoV-Bu	KT228328	2759	132-488/ 357/118	292-1062/ 771/256	1505-2596/ 1092/363	1295-1504/ 210/69	1059-1463/ 405/134	2242-2682/ 441/146	283-807/ 525/174

DC: Downward leaf curling, UC: Upward leaf curling, LE: leaf enation, Vt: Vein thickening, Sev: Severe, nt: nucleotide sequence length, aa: amino acid sequence length

**Table 2:** Pairwise nucleotide identity among complete begomovirus genomes, based on pairwise distance analysis calculated using the Sequence Demarcation Tool.

CLCuD sequence	CLCuD-begomoviruses/strain (nt identity %)												
	CLCuMuV					CLCuKoV					CLCuAlV	CLCuBaV	CLCuGeV
	Rajasthan (Ra)	Hisar (His)	Pakistan (PK)	Faisalabad (Fai)	Hibiscus (Hib)	Kokhran (Ko)	Burewala (Bu)	Shadadpur (Sha)	Layyah (La)	Lucknow (Lu)			
Faz-14	**95–98**	86	93	86	81	87	83	91	86	86	78–81	82	69
Ma-14-3	**97–100**	86	92	86	81	89	82–83	91	86	87	79–81	82	70
Rh-4	**94–97**	85	92	86	80	87	82–83	91	86	85	78–81	82	69
S-9	**95–98**	86	93	87	81	87	82–83	91	87	86	79–81	82	70
Sa-3	**94–95**	86	91	87	80	85	84–85	89	89	83	79–81	81	69
Si-17	**95–97**	86	93	86	81	87	82–83	91	86	86	78–81	82	69
SG-14	**97–100**	86	92	86	81	89	82–83	91	86	87	78–81	83	70
Uf-1	**97–100**	86	92	86	81	89	82–83	91	86	87	78–81	83	70
S-11	84–86	93	90	**97**	83	77	84	84	89	81	80–85	82	69
Hi-3	90–92	89	**100**	90	86	82	81	90	86	81	81–86	83	69
Hi-14	83	81	81	84	74	89	**98–99**	90	93	88	73–76	82	69
Hmg-14	83	81	81	84	74	88	**98–99**	90	92	88	73–75	82	69
Si-14-1	83	81	81	84	74	88	**98–99**	90	92	88	73–75	82	69

Highest percent nucleotide identity of the present sequences is represented by bold font

The present study demonstrated the occurrence of CLCuMuV-Ra, CLCuMuV-PK, CLCuMuV-Fai, and CLCuKoV-Bu stains in NW India, where CLCuMuV-Ra is the most prevalent strain. Earlier, it has been reported that CLCuKoV-Bu strain was dominant population of CLCuD-begomovirus in Punjab and Rajasthan states of India during the years of 2009 to 2010 [4]. After that CLCuMuV has been reported to be associated with CLCuD outbreak in both the states during 2013–2015 [29,30]. Thus, the previous [29,30] and the present studies showed shifting of CLCuD-begomovirus from time to time in NW India, indicating return of CLCuMuV-Ra in NW India, corroborating with the recent report of rebound of CLCuMuV for CLCuD outbreak in Pakistan [22].

### Single betasatellite species CLCuMB is associated with CLCuD outbreak in NW India

Three betasatellite sequences with varying length (1282–1373 nt) were obtained from the CLCuD-begomovirus infected cotton samples. All the sequences had a ~357nt βC1 gene, typical to βC1 gene of other betasatellite, in the complementary sense strand (Table 3). The present betasatellites had 87–92% nt identity among themselves, and they were related to Cotton leaf curl Multan betasatellite (CLCuMB) by 83–95% nt identity. Based on species demarcation cut-off value at ≥78% nt identity proposed by Briddon et al. [60], the present betasatellites are the members of CLCuMB; reveals that occurrence of a single betasatellite species CLCuMB in association with CLCuD outbreak in NW India.

**Table 3:** Genomic properties of CLCuD-begomovirus associated betasatellite and alphasatellite molecules generated in the present study.

Satellite molecules associated with CLCuD sequence	Betasatellite				Alphasatellite			
	Species	Acc No.	Size	ORF βC1 (coordinates/ nt/aa)	Species	Acc No.	Size	ORF Rep (coordinates/ nt/aa)
Ma-14-3 (CLCuMuV-Ra)	CLCuMB	KT228325	1282	194-550/357/118	GDarSLA	KT228319	1353	80-1027/948/315
Rh-4 (CLCuMuV-Ra)					GDarSLA	KM103525	1386	70-1017/948/315
Sa-3 (CLCuMuV-Ra)					GDarSLA	KM103526	1378	70-1017/948/315
Uf-1 (CLCuMuV-Ra)					CrYVMA	KM103524	1382	58-945/888/295
S-11 (CLCuMuV-Fai)	CLCuMB	KM103522	1373	195-551/357/118				
Hi-3 (CLCuMuV-PK)					GDarSLA	KM103523	1374	70-1017/948/315
Si-14-1 (CLCuKoV-Bu)	CLCuMB	KT228326	1335	201-557/357/118	GDarSLA	KT228320	1359	80-1027/948/315

nt: nucleotide sequence length, aa: amino acid sequence length

**Fig 3:**
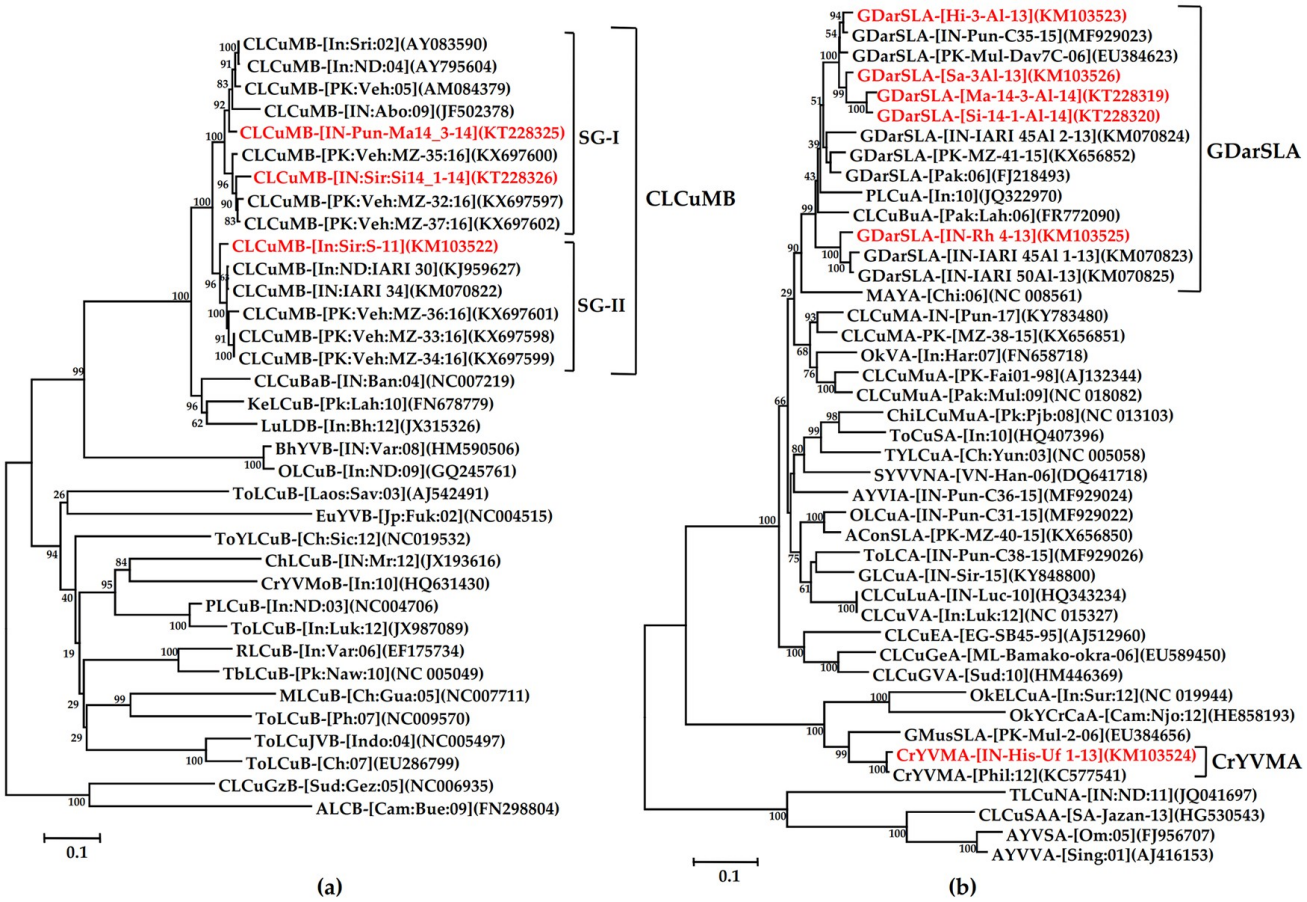
Phylogenetic relationships based on the complete genome of the present betasatellite molecules with other betasatellites (a), and the present alphasatellite molecules with other alphasatellites available in NCBI-GenBank (b). Phylogenetic tree was generated using the Neighbor-Joining (NJ) with 1000 bootstrap iterations in MEGA 6 software. The sequences generated in the present study are represented by the red colour font. CLCuMB, GDarSLA and CrYVMA clades demarcated in the right panel of the figure.

Recently Zubair et al. [22] also identified three types of betasatellites, CLCuMB^Bur^, CLCuMB^Veh^ and CLCuMB^Mul^ in association with CLCuD-begomovirus in Pakistan. In the phylogenetic analysis all the three present betasatellites made one group along with all the other CLCuMB; but upon closer inspection this group was also divided into two subgroups; subgroup- I (SG-I) and -II (SG-II). The SG-I consisted of two betasatellites KT228325 and KT228326, and they showed 93–94% nt identity with both the betasatellites CLCuMB^Bur^ (CLCuMB-PK:Veh:MZ-35:16:KX697600) and CLCuMB^Veh^ (CLCuMB-PK:Veh:MZ-37:16:KX697602). The SG-II consisted of one betasatellite KM103522 and it showed 92% nt identity with CLCuMB^Mul^ (CLCuMB-PK:Veh:MZ-33:16: KX697598) (Fig 3a).

### Alphasatellite species GDarSLA is predominantly associated with CLCuD-begomovirus complex in NW India

Six alphasatellite sequences with varying length of 1353–1386 nt were obtained from the CLCuD-begomovirus infected cotton samples. All the sequences had a typical Rep gene of ~948 nt in the virion-sense strand (Table 3). Sequence analysis showed that the present alphasatellite sequences had 47–95% nt identity among themselves. Five alphasatellite sequences, KM103523, KM103525, KM103526, KT228319 and KT228320 were related to alphasatellite GDarSLA-[IN-Pun-C35-15](MF929023) by 79–98% nt identity. The alphasatellite sequence, KM103524 was related to Croton yellow vein mosaic alphasatellite (CrYVMoA:KC577541) by 95% nt identity. Considering the classification of the family *Alphasatellitidae* using species demarcation cut-off value at ≥89% nt identity proposed by Briddon et al. [31], the present alphasatellite was placed under two alphasatellite species, GDarSLA and CrYVMoA. Corroborating with the pairwise nt identity result, five present alphasatellite sequences were phylogenetically affiliated to GDarSLA and one was to CrYVMoA (Fig 3b). Thus, the present study reveals occurrence of two alphasatellite species GDarSLA and CrYVMA in CLCuD complex in NW India, where GDarSLA is predominant. Earlier, occurrence of seven alphasatellites, GDarSLA, Cotton leaf curl burewala alphasatellite (CLCuBuA), CLCuMuA, Okra leaf curl alphasatellite (OLCuA), Tomato leaf curl alphasatellite (ToLCA), Ageratum yellow vein India alphasatellite (AYVIA) and Gaur leaf curl alphasatellite (GLCuA) have been reported in cotton growing areas of NW India [7,29,30]. However, of them, except GDarSLA and CLCuMuA, others were not considered as species in the recent classification given by Briddon et al. [31]. Therefore, it needs proper classification system for CLCuD associated alphasatellites in order to eliminate the taxonomic ambiguity.

### Recombination is the common phenomenon for evolution of CLCuD-begomovirus variants and CLCuMuV-Ra strain is highly recombinant

**Fig 4:**
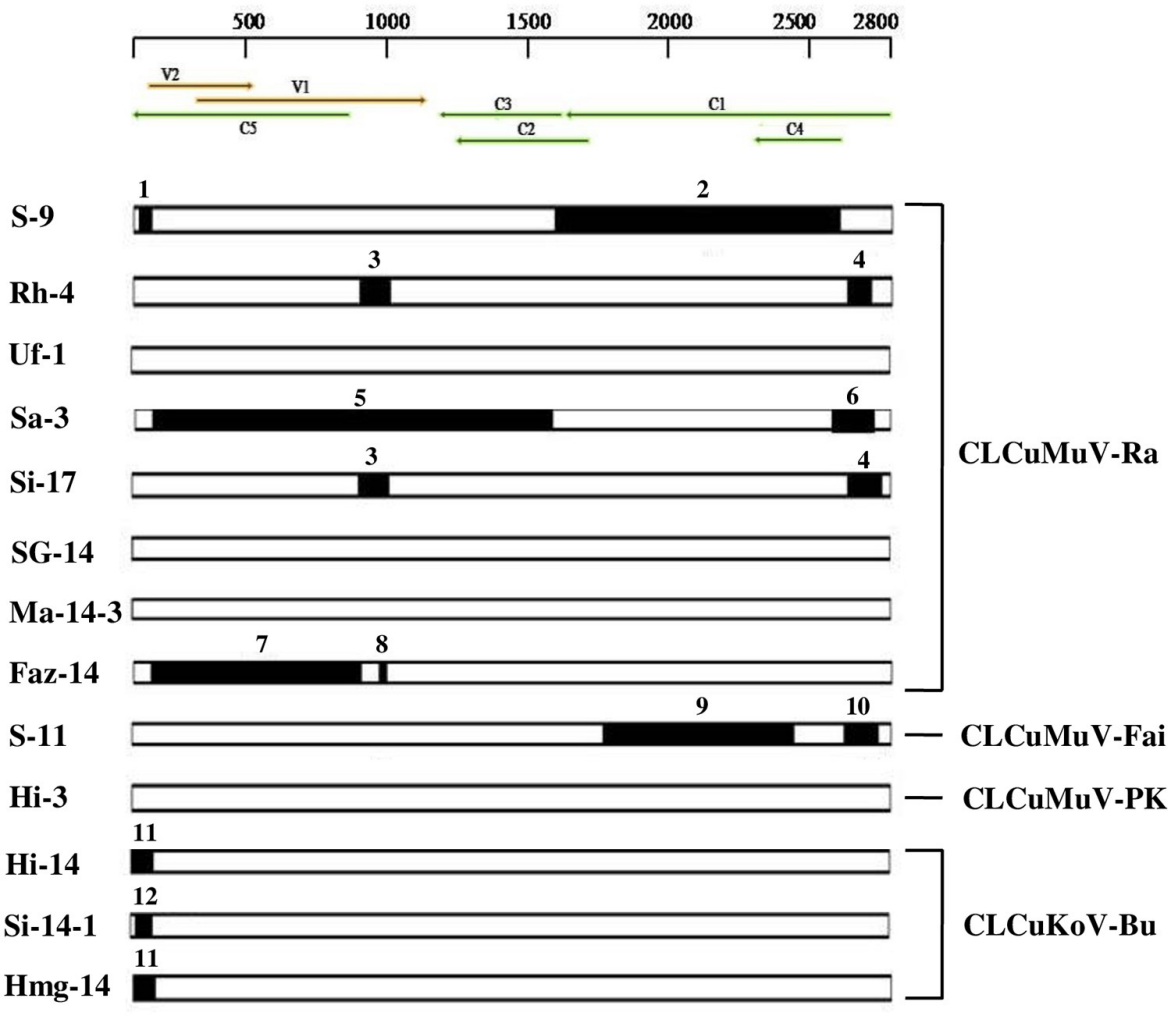
Recombination events identified in the present CLCuD-begomovirus genomes; genetic map of DNA-A is shown at the top of the figure, recombinant fragments are represented by dark colour bars along with minor parent involved in each recombination event represented by the bold number. 1:CLCuMuV- PK/Hib, 2:CLCuMuV-Ra, 3:CLCuBaV, 4:CLCuMuV-Ra/CLCuKoV-Bu, 5:CLCuMuV-Fai, 6:CLCoKoV-Bu, 7:CLCuMuV-Ra, 8:CLCuMuV-Ra, 9:CLCuAlV-Al/CLCoKoV-Bu, 10:CLCoKoV-Bu, 11:CLCuBaV/CYVMV, 12:CLCuMuV-Ra; CLCuD-begomovirus strains marked on the right panel of the figure.

The present CLCuD-begomovirus DNA-A sequences showed clear recombination events. Of 13 present sequences nine were detected as recombinants involving 12 recombination events. Two patterns of recombination were observed, recombination involving (i) coding and IR regions for CLCuMuV-Ra and -Fai strains, and (ii) IR region for CLCuKoV-Bu strain (Table 4; Fig 4). But recombination was not detected for CLCuMuV-PK strain. Inter-species recombination events were common in all the recombinant sequences, as involvement of inter-species donor sequences, CLCuMuV x CLCuBaV/CLCuKoV for CLCuMuV-Ra, CLCuMuV x CLCuAlV/CLCuKoV for CLCuMuV-Fai, and ToLCuNDV/*Croton yellow vein mosaic virus* (CrYVMV)/CLCuKoV x CLCuBaV/CLCuMuV for CLCuKoV-Bu strains were detected. The CLCuMuV-Ra strain was detected as highly recombinant and it has evolved due to inter-species recombination involving multiple begomoviruses, CLCuMuV, CLCuKoV, and CLCuBaV as donor sequences. On the other hand, although CLCuMuV-Ra strain reported from Pakistan is a recombinant but it has evolved due to inter-species recombination involving donor sequences, CLCuMuV and CLCuKoV [22]. Therefore, CLCuMuV-Ra strain of India is distinct from CLCuMuV-Ra of Pakistan. In the present study, they were also separately placed in the phylogenetic tree (Fig 2).

**Table 4:** Recombination analysis of CLCuD-begomovirus genomes using the Recombination Detection Program version 4.66.

CLCuD sequence	Recombination site (region)	Length (nt)	Event number	Major parent x minor parent	Detected by method^a^	Max. P value^b^
Faz-14 (CLCuMuV-Ra)	64–901 (IR,V1,V2)	838	7	CLCuBaV/MeYVMV**x**CLCuMuV-Ra	R,M,B,G,Si	2.72x 10^−9^
	975–1001 (V1)	27	8	CLCoKoV-Bu/CLCuKoV-Lu **x**CLCuMuV-Ra	G,R,M	8.05 x 10^−3^
Rh-4 (CLCuMuV-Ra)	900–1002 (V1)	103	3	CLCuMuV-Ra **x**CLCuBaV	3-S,B,C,M,R	1.72 x 10^−9^
	2632–2714 (IR)	83	4	CLCuMuV-Ra **x** CLCuMuV-Ra/CLCuKoV-Bu	C,M,R,G	4.66 x 10^−5^
S-9 (CLCuMuV-Ra)	16–63 (IR)	48	1	CLCuMuV-Ra **x**CLCuMuV- PK/Hib	R,G,3-S	7.35 x 10^−4^
	1595–2610 (C1, C4)	1016	2	CLCuMuV-Ra **x** CLCuMuV-Ra	3-S,C,Si	1.2 x 10^−2^
Sa-3 (CLCuMuV-Ra)	62–1596 (IR,V1,V2,C1, C2, C3)	1535	5	CLCuKoV-La **x**CLCuMuV-Fai	3-S,C,M	9.8 x 10^−15^
	2586–2723 (IR, C1, C4)	141	6	CLCuMuV-Fai **x**CLCoKoV-Bu	C,M,G	2.1 X 10^−7^
Si-17 (CLCuMuV-Ra)	900–1002 (V1)	103	3	CLCuMuV-Ra **x** CLCuBaV	C,3-S,R	1.71 x 10^−9^
	2642–2752 (IR)	111	4	CLCuMuV-Ra **x**CLCuMuV-Ra	G,M,R	2.8 x 10^−15^
S-11(CLCuMuV-Fai)	1774–2442 (C1, C4)	669	9	CLCuMuV-His **x**CLCuAlV-Al/CLCoKoV-Bu	Si,M,C	9.1 x 10^−41^
	2623–2749 (IR)	127	10	CLCuMuV-His **x**CLCoKoV-Bu	C,R,G	1.53 x 10^−15^
Hi -14 (CLCuKoV-Bu)	1–76 (IR)	76	11	ToLCuNDV/CLCuMuV-Ra **x**CLCuBaV/ CYVMV	R,B,G	2.78x10^-3^
Si-14-1 (CLCuKoV-Bu)	11–75 (IR)	65	12	ToLCuNDV/CYVMV/CLCuKoV-La **x**CLCuMuV-Ra	B,C,G,R	2.78 x 10−^3^
Hmg-14 (CLCuKoV-Bu)	1–76 (IR)	76	11	ToLCuNDV/CLCuBaV/ CLCuMuV-Ra **x**CLCuBaV/CLCuMuV-Ra/CYVMV	R,B,G	2.78x10^-3^

^a^: B Bootscan, C Chimera, G Geneconv, M Maxchi, R RDP, Si Siscan and 3-S 3SEQ implemented in the RDP4,

^b^: highest acceptable P-value cut-off of 0.05 detected the evidences of recombination events among the sequences; *Croton yellow vein mosaic virus*, CYVMV (JN817516), *Cotton leaf curl Bangalore virus*, CLCuBaV (AY705380), *Mesta yellow vein mosaic virus*, MeYVMV (FJ159262) and *Tomato leaf curl New Delhi virus*, ToLCuNDV (KC545812)

The betasatellite and most of the alphasatellites associated with CLCuD-begomovirus are recombinants

All the present betasatellites were found to be recombinants showing five clear recombination events (Table 5). Betasatellite sequences KT228325 and KM103522 showed recombination in βC1 gene and KT228326 in SCR. The SCR region has been considered as a hotspot for recombination in CLCuMB [22]. However, in this study most of the recombination in the present betasatellites was detected in the βC1 gene indicating the coding region is also a hotspot for the recombination of CLCuMB. Of six present alphasatellites tested, four were recombinants involving the Rep gene and the A-rich regions for recombination (Table 5). Interestingly, all the present recombinant alphasatellites are GDarSLA. Alphasatellite sequences KM103526 and KT228319 showed recombination in both the Rep gene and A-rich region, and KM103523 in Rep, and KM103525 in A-rich region.

**Table 5:** Recombination analysis of betasatellite and alphasatellite molecules using Recombination Detection Program, version 4.66.

Satellite molecules	Associated with CLCuD sequence	Recombination site (region)	Event Number	Major parent x minor parent	Detected by method^a^	Max. P value^b^
**Betasatellite**						
KT228325 (CLCuMB)	Ma-14-3 (CLCuMuV-Ra)	17–762 (βC1)	1	ToYLCuB (NC019532) **x**CLCuMB (KT228326*)	R,B,3-S	2.58x10^-7^
		1160–1201 (upstream of SCR)	2	ToLCuB (NC 009570)/PLCuB (NC004706) **x** CLCuGzB(NC006935)	R,G, M	3.12x10^-3^
KM103522 (CLCuMB)	S-11 (CLCuMuV-Fai)	129–870 (βC1)	3	LuLDB (JX315326)/ToLCuJVB (NC005497)/ CLCuMB (KT228326*) **x** CLCuMB/ CrYVMoB (HQ631430)	B, M, 3-S	5.67x10^-4^
		948–1028 (A-rich)	4	CLCuMB (IARI-30)/CLCuMB ((KT228326*)/CLCuMB**x**CrYVMoB (HQ631430)/ToLCuB (NC 009570)/ LuLDB (JX315326)	R, B, C	1.38 x 10^−3^
KT228326 (CLCuMB)	Si-14-1 (CLCuKoV-Bu)	1–110 (SCR)	5	ALCB (NC012557) **x**CLCuMB	G,M	1.14x10^-9^
**Alphasatellite**						
KM103525 (GDarSLA)	Rh-4 (CLCuMuV-Ra)	1030–1111 (A-rich)	1	GDarSLA (FJ218493) **x**MaYA (NC008561)/CLCuMuA (NC018082) /CLCuBuA (FR772090)	R,M,C	1.7x10^-9^
KM103523 (GDarSLA)	Hi-3 (CLCuMuV-PK)	1–996 (Rep)	6	AYVSA (FJ956707)/OkVA (FN658718)/TLCuNA (JQ041697) **x**GDarSLA (KM103525^ψ^)/ GDarSLA (KM103526^ω^)	B,G,3-S	5.01x10^-8^
KT228319 (GDarSLA)	Ma-14-3 (CLCuMuV-Ra)	1–1025 (Rep)	4	TLCuNA (JQ041697)/GDarSLA (KM103525 ^ψ^) /MaYA (NC008561) **x** GDarSLA (KT228320*)	G,B,3-S	2.76x10^-10^
		1028–1086 (A-rich)	5	GDarSLA (KT228320*) **x**MaYA (NC008561)/CLCuBuA (FR772090)	R,M,C	4.72x10^-16^
KM103526 (GDarSLA)	Sa-3 (CLCuMuV-Ra)	64–639 (Rep)	2	CLCuBuA (IARI-45-2)/CLCuBuA (FR772090)/CLCuGVA (HM446369) **x**GDarSLA (FJ218493)/ GDarSLA (KM103523^π^)	R,G,3-S	2.1x10^-2^
		1185–1263 (A-rich)	3	GDarSLA (FJ218493)/ GDarSLA (KM103523^π^) **x**CLCuMuA (NC018082)	B,M,C	5.9x10^-4^

^a^:B Bootscan, C Chimera, G Geneconv, M Maxchi, R RDP, S Siscan and 3-S 3SEQ implemented in the RDP4,

^b^: highest acceptable P-value cut-off of 0.05 detected evidences of recombination events among the sequences; CLCuD-begomovirus represented by special character superscript to the Accession no’s. of associated betasatellite and alphasatellite molecule; Si-14-1 (CLCuKoV-Bu) by *, Hi-3 (CLCuMuV-PK) by ^π^, Rh-4 (CLCuMuV-Ra) by ^ψ^ and Sa-3 (CLCuMuV-Ra) by ^ω^; *Tomato yellow leaf curl betasatellite* (ToYLCuB), *Tomato leaf curl betasatellite* (ToLCuB), *Papaya leaf curl betasatellite* (PLCuB), *Cotton leaf curl Gezira betasatellite* (CLCuGzB), *luffa leaf distortionbetasatellite (*LuLDB), *Tomato leaf curl Joydebpur virus* (ToLCuJVB), *Croton yellow vein mosaic betasatellite (*CrYVMoB), *Ageratum leaf curl betasatellite* (ALCB), *Malvastrum yellow mosaic alphasatellite* (MaYA), *Cotton leaf curl Gezira alphasatellite* (CLCuGVA), Tomato leaf curl New Delhi alphasatellite (TLCuNA), *Ageratum yellow vein Singapore alphasatellite* (AYVSA),Okra virus alphasatellite (OkVA)

The evolution of recombinant betasatellite has been reported to be associated with resistance breaking in cotton against CLCuD [37,38]. Recently, recombinant CLCuMB^Bur^ in association with CLCuKoV-Bu strain has been reported to cause resistance breaking in cotton varieties in Pakistan [61]. Thus, the present study indicated that recombinant CLCuMB may be the cause of the increased CLCuD outbreak in NW India.

### CLCuKoV-Bu strain of NW India contains truncated and extended ORFs

In closer inspection, a significant sequence variation was observed in C2, C4, and C5 ORFs among the present CLCuMuV strains (-Ra, -Fai, -Pak) and CLCuKoV-Bu strain. The ORF C2 of all the present CLCuMuV strains encodes full length TrAP of 150 amino acid (aa) (wild type), whereas all the CLCuKoV-Bu strains encode a truncated TrAP of 69 aa (mutant) (Table 1). The ORF C4 of the present CLCuMuV strains encodes full length protein of 100 aa (wild type), whereas all the present CLCuKoV-Bu strain encode an extended protein of 146 aa (mutant) (Table 1). The C5 ORF of the present CLCuMuV strains encode full length protein of 243 aa (wild type), whereas all the CLCuKoV-Bu strains encode a truncated protein of 174 aa (mutant) (Table 1).

TrAP encoded by C2 ORF is a multi-functional protein and it functions as a pathogenicity determinant [62] and acts as suppressor of PTGS [63]. CLCuKoV-Bu strain which was dominant in Pakistan during the year 2000 and onward encoded truncated TrAP of 35 aa [38,43]. The TrAP of the present CLCuKoV-Bu strain is truncated (69 aa) but larger in length than that of CLCuKoV-Bu of Pakistan, indicating differences in suppressor activities between CLCuKoV-Bu of Pakistan and India. The truncated TrAP of CLCuKoV-Bu has been reported to overcome resistance in cotton [38,43,64]. Earlier, CLCuMuV has been shown to encode 100 aa C4 proteins, whereas CLCuKoV-Bu to encode an extended protein of 181 aa [4]. Thus, the previous [4] and the present study show that the C4 ORF of CLCuKoV-Bu has periodically undergone significant changes with respect to amino acid encoded by ORF C4. Earlier, majorities of CLCuKoV-Bu and some of CLCuMuV-Ra strains of NW India showed the presence of C5 ORF in their genome, but this ORF is not reported in the genome of the cotton-infecting begomoviruses in Pakistan [4,7].

## Conclusions

The present study reveals that CLCuD is a major constraint in the cultivation of cotton and the disease outbreak has been changing constantly in year to year and area to area in NW India. The CLCuMuV-Ra strain is identified as the most predominant begomovirus, which is evolved from inter-species recombination. To a much lesser extent, the CLCuKoV-Bu, CLCuMuV-PK, CLCuMuV-Fai were identified from symptomatic cotton plants of NW India. Single betasatellite species CLCuMB, and more numbers of alphasatellite species, GDarSLA and CrYVMoA are associated with the CLCuD complex. Therefore, it is concluded that the recombinant CLCuD-begomovirus, particularly recombinant CLCuMuV-Ra, in association with recombinant CLCuMB betasatellite are the important causes for outbreak of CLCuD in NW India in present condition.

## Supporting information

S1 Table CLCuD incidence in cotton growing areas of Haryana, Punjab and Rajasthan states of northwest India for three successive years of 2012 to 2014. (DOCX)

S2 Table Percent nucleotide identity matrix of mtCO1 gene of present whitefly (*Bemicia tabaci*) with other whitefly based on nucleotide sequence. (DOCX)

S3 Table Pathogenicity test of CLCuD field isolates through whitefly species Asia II 1 inoculation in greenhouse. (DOCX)

S1 Fig Phylogenetic relationships based on nucleotide sequence of mtCOI gene of whitefly (*Bemicia tabaci)* population with other whitefly population available in the GenBank. Phylogenetic Neighbor-Joining (NJ) tree, reconstructed using MEGA 6 software, with 1000 bootstrap iterations. The sequences generated in the present study are represented by red colour font and the Asia II 1 clade demarcated in the right panel of the figure.
(TIF)
