# Supplementary material for: Dominance of recombinant cotton leaf curl Multan-Rajasthan virus associated with cotton leaf curl disease outbreak in northwest India
Source: PLoS One. 2020 Apr 22;15(4):e0231886. doi: 10.1371/journal.pone.0231886 (PMC7176085; doi:10.1371/journal.pone.0231886)
Supplement: S1 Table — (DOCX) [file pone.0231886.s001.docx]

**S1 Table.** CLCuD incidence in cotton growing areas of Haryana, Punjab and Rajasthan states of northwest India for three successive years of 2012 to 2014

| **District** | **Geographic coordinates** | | **2012** | | | **2013** | | | **2014** | | |
| --- | --- | --- | --- | --- | --- | --- | --- | --- | --- | --- | --- |
|  | **Latitude (°N)** | **Longitude (°E)** | **No. of field surveyed** | **Disease* incidence (%) range** | **Average disease incidence (%)** | **No. of field surveyed** | **Disease* incidence (%) range** | **Average disease incidence (%)** | **No. of field surveyed** | **Disease* incidence (%) range** | **Average disease incidence (%)** |
| Haryana state | | | | | | | | | | | |
| Fatehabad | 29.51 | 75.45 | 9 | 9.5-59 .5 | 32.4 | 16 | 59.5-97 .5 | 80.8 | 15 | 35.0 -84.6 | 58.3 |
| Hisar | 29.14 | 75.72 | 13 | 17.2-88.6 | 37.5 | 19 | 49.5-100 | 80.5 | 24 | 25.1-66.3 | 43.4 |
| Rohtak | 28.89 | 76.60 | 6 | 20.5-70.8 | 32.6 | 7 | 43.3-100 | 74.7 | 12 | 30.4-60.0 | 49 .0 |
| Sirsa | 29.53 | 75.01 | 12 | 17.5-65.2 | 28.2 | 20 | 47.5-95.2 | 73.9 | 19 | 35.0 -81.2 | 48.0 |
| Overall Haryana | | | 40 | 9.5-88.6 | 32.7 | 62 | 43.3-100 | 77.5 | 70 | 25.1-84.6 | 49.6 |
| Punjab state | | | | | | | | | | | |
| Bathinda | 30.21 | 74.94 | 7 | 27.5 -87.5 | 58.5 | 9 | 27.5 -53.8 | 40.4 | 12 | 36.9-62.2 | 48.0 |
| Faridkot | 30.67 | 74.75 | 2 | 36.4-79.5 | 53..8 | 3 | 77.8-98.2 | 87.5 | 4 | 90.2-100 | 97.5 |
| Fazilka | 30.40 | 74.02 | 15 | 38.5-72.8 | 54.3 | 27 | 13.5-60.1 | 38.6 | 28 | 7.5-67.5 | 45.7 |
| Mansa | 29.99 | 75.39 | 3 | 15.5 -44.5 | 38.6 | 4 | 45.5 -54.5 | 50 .0 | 4 | 15.2 -64.8 | 40 .0 |
| Overall Punjab | | | 27 | 15.5-87.5 | 51.3 | 43 | 13.5-98.2 | 54.1 | 48 | 7.5-100 | 57.8 |
| Rajasthan state | | | | | | | | | | | |
| Hanumangarh | 29.61 | 74.29 | 8 | 7.0-42.7 | 18.7 | 16 | 35.3-70.0 | 55.0 | 16 | 2.5-12.5 | 7.0 |
| Sri Ganganagar | 29.90 | 73.87 | 10 | 10.0-60.5 | 27.8 | 16 | 34.8-97.5 | 63.4 | 18 | 2.9-25.0 | 10.6 |
| Overall Rajasthan | | | 18 | 7.0-60.5 | 28.6 | 32 | 34.8-97.5 | 59.2 | 34 | 2.5-25.0 | 8.9 |
| Overall northwest India | | | 85 | 7.0-88.6 | 37.5 | 137 | 13.5-100 | 63.6 | 152 | 2.5-100 | 38.8 |

*: observation of 100-200 plants, with three replications, randomly per cotton field infected by CLCuD
