# Supplementary material for: Dominance of recombinant cotton leaf curl Multan-Rajasthan virus associated with cotton leaf curl disease outbreak in northwest India
Source: PLoS One. 2020 Apr 22;15(4):e0231886. doi: 10.1371/journal.pone.0231886 (PMC7176085; doi:10.1371/journal.pone.0231886)
Supplement: S2 Table — (DOCX) [file pone.0231886.s002.docx]

**S2 Table.** Percent nucleotide identity matrix of mtCO1 gene of present whitefly (*Bemicia tabaci*) with other whitefly based on nucleotide sequence

| Whitefly sequences | MN329161 [India:Har-Sir:AsiaII_1] | MN329161 [India:Har-Sir:AsiaII_1] | MN329161 [India:Har-Sir:AsiaII_1] | FJ802389[Syria:Hims:AsiaII 1] | AJ510058[Pakistan:Punjab:AsiaII 1] | GU585369[Pakistan:Faisalabad:AsiaII 1] | HM137326[China:Guangdang:AsiaII 1] | AJ867557[China:Zhejiang:AsiaII 1] | DQ174519[Taiwan:Chiayi:AsiaII 1] | EU192047[China:Guangxi:AsiaII1] | GU585372[Pakistan:Punjab:AsiaII 1] | AY686088[China:Jiangsu:AsiaII 2] | EU192045[China:Guangxi:AsiaII 3] | DQ309074[China:Zhejiang:AsiaII 3] | AY686083[China:Guangdong:AsiaII 4] | AJ748376[India:Karnataka:AsiaII 5] | AF418666[India:Karnataka:AsiaII 5] | HQ916813[China:Yunnan:AsiaII 6] | DQ174520[Taiwan:AsiaII 6] | DQ116650[India:Andhra Pradesh:AsiaII 7] | GQ281720[India:Gujarat:AsiaII 7] | AJ748358[India:Karnataka:Asia:II 8] | AJ748374[India:Karnataka:Asia:II 8] | HM137313[China:Hunan:AsiaII 9] | HM137345[China:Hunan:AsiaII 9] | HM137356[China:Guangdong:AsiaII 10] | HM137339[China:Guangdong:AsiaII 10] | HM590147[India:Asia:II 11] | HM590146[India:Asia:II 11] | HM137315[China:Sichuan:China1] | AY686085[China:Hunan:China1] | HQ916820[China:Yunnan:China2] | EU192050[China:Yunnan:China3] |
| --- | --- | --- | --- | --- | --- | --- | --- | --- | --- | --- | --- | --- | --- | --- | --- | --- | --- | --- | --- | --- | --- | --- | --- | --- | --- | --- | --- | --- | --- | --- | --- | --- | --- |
| MN329161 [India:Har-Sir:AsiaII_1] | 100 | 99 | 99 | 89 | 87 | 90 | 85 | 94 | 82 | 92 | 93 | 80 | 84 | 81 | 76 | 81 | 83 | 76 | 75 | 83 | 74 | 79 | 79 | 75 | 75 | 74 | 75 | 81 | 81 | 73 | 74 | 73 | 79 |
| MN329162 [India:Raj-SGNR:AsiaII_1] |  | 100 | 99 | 89 | 88 | 90 | 85 | 95 | 83 | 92 | 93 | 80 | 84 | 81 | 77 | 82 | 83 | 76 | 75 | 83 | 75 | 80 | 79 | 75 | 76 | 74 | 75 | 82 | 81 | 73 | 74 | 73 | 80 |
| MN329163[India:Pun-Bha:AsiaII_1] |  |  | 100 | 89 | 88 | 90 | 86 | 95 | 83 | 92 | 93 | 80 | 84 | 81 | 76 | 82 | 83 | 76 | 75 | 83 | 74 | 80 | 80 | 76 | 76 | 75 | 75 | 82 | 81 | 74 | 74 | 74 | 80 |
| FJ802389[Syria:Hims:AsiaII 1] |  |  |  | 100 | 94 | 92 | 90 | 93 | 91 | 94 | 90 | 89 | 83 | 85 | 84 | 88 | 88 | 81 | 82 | 87 | 81 | 86 | 86 | 80 | 80 | 79 | 79 | 86 | 85 | 77 | 81 | 77 | 82 |
| AJ510058[Pakistan:Punjab:AsiaII 1] |  |  |  |  | 100 | 96 | 95 | 92 | 93 | 92 | 94 | 90 | 81 | 83 | 85 | 86 | 86 | 85 | 84 | 85 | 83 | 84 | 84 | 84 | 84 | 83 | 83 | 84 | 84 | 81 | 82 | 81 | 79 |
| GU585369[Pakistan:Faisalabad:AsiaII 1] |  |  |  |  |  | 100 | 93 | 92 | 90 | 90 | 96 | 87 | 81 | 83 | 83 | 84 | 86 | 83 | 81 | 85 | 80 | 82 | 82 | 82 | 82 | 81 | 81 | 84 | 83 | 79 | 80 | 79 | 78 |
| HM137326[China:Guangdang:AsiaII 1] |  |  |  |  |  |  | 100 | 90 | 94 | 88 | 91 | 86 | 78 | 81 | 81 | 83 | 83 | 88 | 84 | 83 | 80 | 81 | 80 | 87 | 87 | 86 | 86 | 81 | 81 | 84 | 78 | 84 | 76 |
| AJ867557[China:Zhejiang:AsiaII 1] |  |  |  |  |  |  |  | 100 | 87 | 97 | 91 | 84 | 87 | 85 | 80 | 86 | 87 | 80 | 78 | 86 | 77 | 83 | 83 | 79 | 79 | 78 | 78 | 85 | 85 | 76 | 77 | 76 | 83 |
| DQ174519[Taiwan:Chiayi:AsiaII 1] |  |  |  |  |  |  |  |  | 100 | 87 | 88 | 91 | 76 | 79 | 86 | 82 | 81 | 84 | 90 | 81 | 85 | 80 | 79 | 83 | 83 | 82 | 83 | 79 | 79 | 80 | 83 | 81 | 76 |
| EU192047[China:Guangxi:AsiaII1] |  |  |  |  |  |  |  |  |  | 100 | 88 | 85 | 86 | 84 | 80 | 86 | 86 | 79 | 78 | 85 | 78 | 84 | 84 | 78 | 78 | 78 | 78 | 84 | 84 | 75 | 77 | 76 | 85 |
| GU585372[Pakistan:Punjab:AsiaII 1] |  |  |  |  |  |  |  |  |  |  | 100 | 85 | 79 | 82 | 81 | 83 | 84 | 82 | 80 | 84 | 79 | 81 | 80 | 80 | 81 | 80 | 80 | 82 | 82 | 78 | 79 | 78 | 76 |
| AY686088[China:Jiangsu:AsiaII 2] |  |  |  |  |  |  |  |  |  |  |  | 100 | 83 | 86 | 95 | 84 | 83 | 81 | 86 | 83 | 85 | 83 | 83 | 84 | 84 | 82 | 82 | 83 | 82 | 80 | 87 | 80 | 80 |
| EU192045[China:Guangxi:AsiaII 3] |  |  |  |  |  |  |  |  |  |  |  |  | 100 | 96 | 86 | 84 | 85 | 78 | 76 | 84 | 76 | 85 | 84 | 85 | 85 | 81 | 81 | 86 | 86 | 77 | 77 | 77 | 84 |
| DQ309074[China:Zhejiang:AsiaII 3] |  |  |  |  |  |  |  |  |  |  |  |  |  | 100 | 88 | 86 | 88 | 81 | 78 | 87 | 78 | 87 | 87 | 87 | 88 | 83 | 83 | 89 | 89 | 79 | 79 | 79 | 82 |
| AY686083[China:Guangdong:AsiaII 4] |  |  |  |  |  |  |  |  |  |  |  |  |  |  | 100 | 82 | 82 | 80 | 84 | 81 | 84 | 82 | 81 | 85 | 85 | 81 | 81 | 82 | 81 | 78 | 86 | 79 | 78 |
| AJ748376[India:Karnataka:AsiaII 5] |  |  |  |  |  |  |  |  |  |  |  |  |  |  |  | 100 | 98 | 86 | 85 | 88 | 80 | 88 | 88 | 81 | 81 | 80 | 81 | 87 | 87 | 80 | 81 | 80 | 82 |
| AF418666[India:Karnataka:AsiaII 5] |  |  |  |  |  |  |  |  |  |  |  |  |  |  |  |  | 100 | 87 | 84 | 89 | 80 | 87 | 87 | 82 | 82 | 81 | 82 | 89 | 89 | 81 | 80 | 80 | 82 |
| HQ916813[China:Yunnan:AsiaII 6] |  |  |  |  |  |  |  |  |  |  |  |  |  |  |  |  |  | 100 | 92 | 83 | 80 | 81 | 81 | 87 | 87 | 88 | 88 | 82 | 81 | 86 | 79 | 86 | 76 |
| DQ174520[Taiwan:AsiaII 6] |  |  |  |  |  |  |  |  |  |  |  |  |  |  |  |  |  |  | 100 | 81 | 85 | 79 | 79 | 83 | 83 | 83 | 83 | 78 | 78 | 82 | 84 | 81 | 76 |
| DQ116650[India:Andhra Pradesh:AsiaII 7] |  |  |  |  |  |  |  |  |  |  |  |  |  |  |  |  |  |  |  | 100 | 88 | 87 | 86 | 82 | 82 | 82 | 82 | 88 | 87 | 79 | 79 | 80 | 81 |
| GQ281720[India:Gujarat:AsiaII 7] |  |  |  |  |  |  |  |  |  |  |  |  |  |  |  |  |  |  |  |  | 100 | 79 | 79 | 79 | 79 | 79 | 79 | 78 | 78 | 77 | 82 | 78 | 76 |
| AJ748358[India:Karnataka:Asia:II 8] |  |  |  |  |  |  |  |  |  |  |  |  |  |  |  |  |  |  |  |  |  | 100 | 99 | 82 | 82 | 82 | 82 | 89 | 89 | 79 | 80 | 79 | 84 |
| AJ748374[India:Karnataka:Asia:II 8] |  |  |  |  |  |  |  |  |  |  |  |  |  |  |  |  |  |  |  |  |  |  | 100 | 82 | 82 | 82 | 82 | 89 | 89 | 79 | 80 | 79 | 84 |
| HM137313[China:Hunan:AsiaII 9] |  |  |  |  |  |  |  |  |  |  |  |  |  |  |  |  |  |  |  |  |  |  |  | 100 | 100 | 90 | 90 | 83 | 83 | 86 | 79 | 86 | 77 |
| HM137345[China:Hunan:AsiaII 9] |  |  |  |  |  |  |  |  |  |  |  |  |  |  |  |  |  |  |  |  |  |  |  |  | 100 | 90 | 90 | 83 | 82 | 85 | 79 | 86 | 77 |
| HM137356[China:Guangdong:AsiaII 10] |  |  |  |  |  |  |  |  |  |  |  |  |  |  |  |  |  |  |  |  |  |  |  |  |  | 100 | 100 | 82 | 82 | 87 | 79 | 87 | 77 |
| HM137339[China:Guangdong:AsiaII 10] |  |  |  |  |  |  |  |  |  |  |  |  |  |  |  |  |  |  |  |  |  |  |  |  |  |  | 100 | 83 | 82 | 87 | 80 | 88 | 78 |
| HM590147[India:Asia:II 11] |  |  |  |  |  |  |  |  |  |  |  |  |  |  |  |  |  |  |  |  |  |  |  |  |  |  |  | 100 | 100 | 80 | 79 | 81 | 82 |
| HM590146[India:Asia:II 11] |  |  |  |  |  |  |  |  |  |  |  |  |  |  |  |  |  |  |  |  |  |  |  |  |  |  |  |  | 100 | 80 | 79 | 81 | 82 |
| HM137315[China:Sichuan:China1] |  |  |  |  |  |  |  |  |  |  |  |  |  |  |  |  |  |  |  |  |  |  |  |  |  |  |  |  |  | 100 | 91 | 96 | 78 |
| AY686085[China:Hunan:China1] |  |  |  |  |  |  |  |  |  |  |  |  |  |  |  |  |  |  |  |  |  |  |  |  |  |  |  |  |  |  | 100 | 88 | 81 |
| HQ916820[China:Yunnan:China2] |  |  |  |  |  |  |  |  |  |  |  |  |  |  |  |  |  |  |  |  |  |  |  |  |  |  |  |  |  |  |  | 100 | 79 |
| EU192050[China:Yunnan:China3] |  |  |  |  |  |  |  |  |  |  |  |  |  |  |  |  |  |  |  |  |  |  |  |  |  |  |  |  |  |  |  |  | 100 |
