# Supplementary material for: Dominance of recombinant cotton leaf curl Multan-Rajasthan virus associated with cotton leaf curl disease outbreak in northwest India
Source: PLoS One. 2020 Apr 22;15(4):e0231886. doi: 10.1371/journal.pone.0231886 (PMC7176085; doi:10.1371/journal.pone.0231886)
Supplement: S3 Table — (DOCX) [file pone.0231886.s003.docx]

**S3 Table.** Pathogenicity test of CLCuD field isolates through whitefly species Asia II 1 inoculation in greenhouse

| **Isolate (source)** | **Cotton cv. inoculated** | **No. Pl. infected / No. Pl. inoculated (%)** | **Days taken for symptom appearance** | **Symptoms induced** |
| --- | --- | --- | --- | --- |
| Faz 14 (Fazilka) | RST-9 | 4/5 (80) | 10-14 | UC, St, Vt |
| Hmg 14 (Hanumangarh) | RST-9 | 4/5 (80) | 15-20 | UC, Vt |
| Uf-1 (Hisar) | RST-9 | 5/5 (100) | 15-28 | DC, UC, St, Vt |
| Ma14-3 (Mansa) | RST-9 | 5/5 (100) | 15-20 | DC, UC, Vt |
| S9 (Sirsa) | RST-9 | 4/5 (80) | 12-14 | UC, Vt |
| SG-14 (Sri Ganganagar) | RST-9 | 5/5 (100) | 15-24 | DC, UC, St, Vt |

DC: Downward leaf curling, UC: Upward leaf curling, Vt: Vein thickening, St: Stunting. Virus sources were inoculated on cotton cv. RST-9; test plants were inoculated through whitefly at cotyledonary stage
