# Supplementary figures and images for: Dominance of recombinant cotton leaf curl Multan-Rajasthan virus associated with cotton leaf curl disease outbreak in northwest India
Source: PLoS One. 2020 Apr 22;15(4):e0231886. doi: 10.1371/journal.pone.0231886 (PMC7176085; doi:10.1371/journal.pone.0231886)

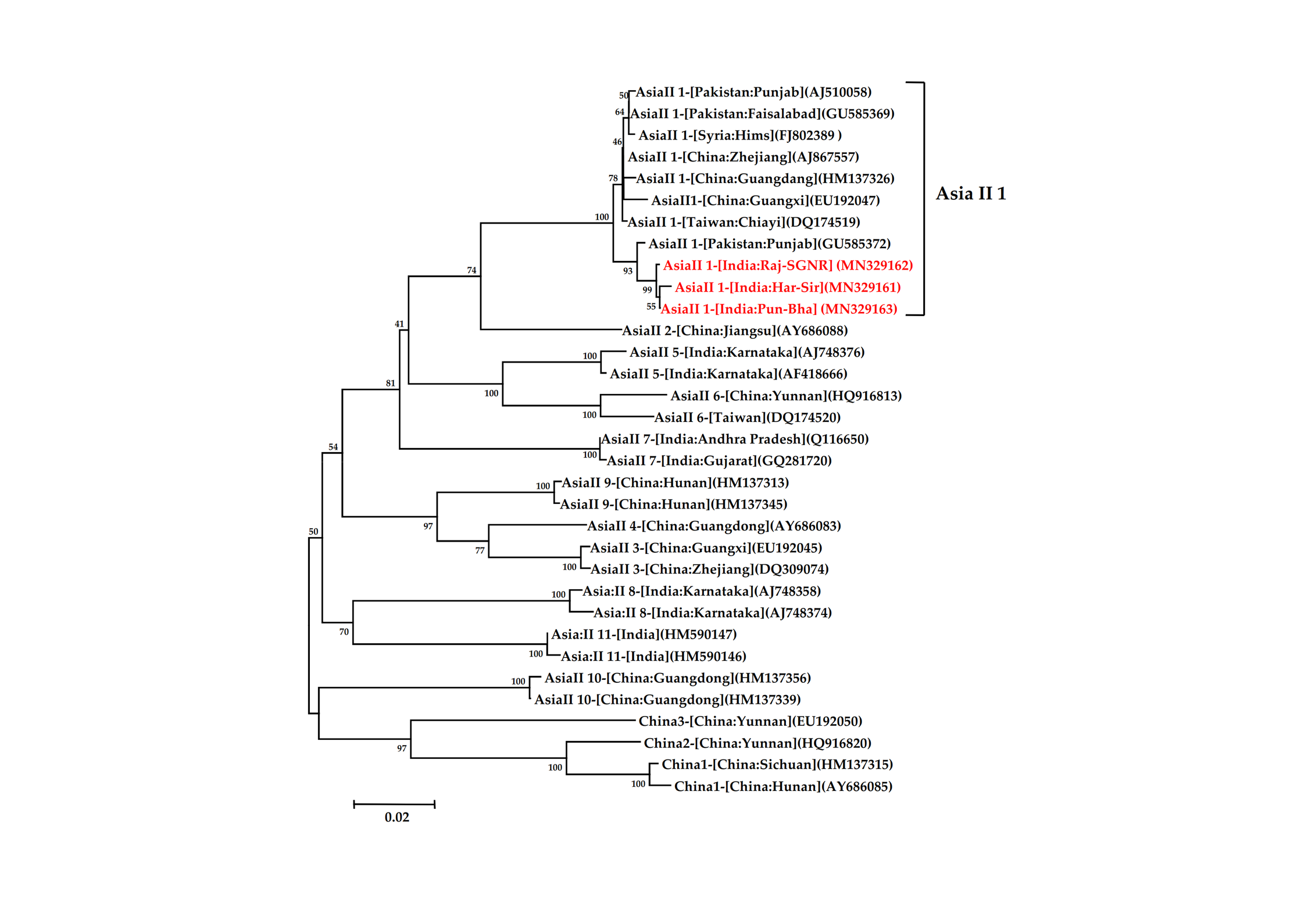

Supplement: S1 Fig — Phylogenetic Neighbor-Joining (NJ) tree, reconstructed using MEGA 6 software, with 1000 bootstrap iterations. The sequences generated in the present study are represented by red colour font and the Asia II 1 clade demarcated in the right panel of the figure. (TIF) [file pone.0231886.s004.tif]
